# Construction of hollow mesoporous silica nanoreactors for enhanced photo-oxidations over Au-Pt catalysts

**DOI:** 10.1093/nsr/nwaa080

**Published:** 2020-04-24

**Authors:** Hao Tian, Jinhui Zhao, Xinyao Wang, Lizhuo Wang, Hao Liu, Guoxiu Wang, Jun Huang, Jian Liu, G Q (Max) Lu

**Affiliations:** State Key Laboratory of Catalysis, Dalian Institute of Chemical Physics (DICP), Chinese Academy of Sciences, Dalian 116023, China; Centre for Clean Energy Technology, School of Mathematical and Physical Sciences, Faculty of Science, University of Technology Sydney, Sydney, NSW 2007, Australia; Laboratory for Catalysis Engineering, School of Chemical and Biomolecular Engineering, Sydney Nano Institute, The University of Sydney, Sydney, NSW 2006, Australia; State Key Laboratory of Catalysis, Dalian Institute of Chemical Physics (DICP), Chinese Academy of Sciences, Dalian 116023, China; Laboratory for Catalysis Engineering, School of Chemical and Biomolecular Engineering, Sydney Nano Institute, The University of Sydney, Sydney, NSW 2006, Australia; Centre for Clean Energy Technology, School of Mathematical and Physical Sciences, Faculty of Science, University of Technology Sydney, Sydney, NSW 2007, Australia; Centre for Clean Energy Technology, School of Mathematical and Physical Sciences, Faculty of Science, University of Technology Sydney, Sydney, NSW 2007, Australia; Laboratory for Catalysis Engineering, School of Chemical and Biomolecular Engineering, Sydney Nano Institute, The University of Sydney, Sydney, NSW 2006, Australia; State Key Laboratory of Catalysis, Dalian Institute of Chemical Physics (DICP), Chinese Academy of Sciences, Dalian 116023, China; DICP-Surrey Joint Centre for Future Materials, Department of Chemical and Process Engineering and Advanced Technology Institute, University of Surrey, Guildford, Surrey GU2 7XH, UK; University of Surrey, Guildford, Surrey GU2 7XH, UK

**Keywords:** nanoreactors, photocatalytic oxidation, metal nanoparticles, mesoporous materials, surface plasmonic resonance

## Abstract

It is highly desirable to design hollow structures with multi-scale functions by mimicking cells for the construction of micro/nanoreactors. Herein, we report the construction of hollow-structured submicrometer-photoreactors with bimetallic catalysts loaded within mesoporous silicas. The synthesis parameters are optimized to study the evolution of hollow structure through hydrothermal treatment and an ‘adhesive-contraction’ formation mechanism is proposed. AuPt@HMZS catalysts exhibited a broader absorbance region under visible light and the adsorption edge displayed a red-shift, indicating the strong metal–metal interactions at the alloy interface. The reaction performance of the coupled Au-Pt catalysts can be tuned to achieve excellent catalytic activity in cinnamyl alcohol oxidation to cinnamic acid for 3.1 mmol g^−1^ with 99% selectivity. The proposed strategy to build hollow structures as multifunctional micro/nanoreactors is promising for the design of high-performance and sustainable catalysts for chemical synthesis.

## Teaser

Taking advantage of the unique structures and spatial catalyst location, hollow-structured submicrometer-photoreactors achieve high catalytic activity and selectivity in the process of photocatalytic oxidation.

## INTRODUCTION

Oxidation of primary alcohols to carboxylic acids is of importance in both organic chemistry and the chemical industry because the oxidation products can be used to prepare various pharmaceuticals and useful chemicals [1,2]. The photocatalytic oxidation process has been considered as a green and sustainable technology for achieving the selective oxidation under ambient conditions with irradiation from solar light [3]. To develop superior photocatalysts with a broad range of light absorption and efficient electron-hole separation, surface modification with metal nanoparticles such as Au and Pt allow for the fast transfer of photoexcited electrons to the surface active sites [4,5]. Therefore, bimetallic Au and Pt catalysts would be desirable by combining the advantages of both surface plasmonic resonance (SPR) effect on Au and activation effect on Pt to further enhance the efficiency for catalytic oxidation under visible light irradiation.

Hollow-structured materials have shown great potential in a variety of applications, including catalysis, drug release and delivery, and energy storage and conversion [6–13]. In particular, hollow-structured materials exhibit some outstanding properties for photocatalytic reactions. Multiple light scattering can be generated within a hollow void to enhance the light-harvesting process [6,9,11,14]. The transport distance among charge carriers can be reduced to facilitate electron-hole transportation [15]. High specific surface area and discrete voids afford abundant accessible surface sites and immobilization of reactive centers for catalytic reactions [16,17]. The uniform channels are excellent at facilitating the reactant diffusion and mass transfer. More reactant molecules can be adsorbed and concentrated within the hollow structure to accelerate reactions [18,19]. To achieve hollow-structured particles, hard template and soft template are employed as the most popular methods. Both methods involve the removal of templates through calcination at high temperature or selective dissolution in specific solvents. However, it remains a great challenge to develop a facile and mild synthetic method to simultaneously create an efficient hollow photocatalytic nanoreactor with ordered porous channels on the shell, well-controlled metal location, broad-spectrum utilization and well-controlled mass transfer and diffusion.

Herein, we report a spontaneous phase transformation of core-shell structured zeolitic imidazolate frameworks (ZIF)-8@SiO_2_ to hollow-structured zinc silica composites under mild hydrothermal conditions. Coating a layer of SiO_2_ on the surface of ZIF-8 and subsequent hydrothermal treatments generates a hollow ‘nanoreactor’, namely, hollow mesoporous zinc silica (HMZS). Additionally, this method could be extended to control the shell thickness for understanding the evolution of the hollow structure. Besides, Au, Pt metal and Au/Pt metal alloy nanoparticles can be successfully encapsulated within the hollow structure. The photocatalytic activity of the obtained AuPt catalysts is much higher than that of monometallic counterparts, indicating the electron-hole recombination can be more effectively suppressed. These distinctive features of the AuPt@HMZS catalysts are concluded as follows: (i) the hollow void between AuPt particles and shell can provide a homogeneous chemical microenvironment for photocatalytic oxidation reaction; (ii) the formation of hollow structure can enhance the light-harvesting process through multiple light scattering; (iii) the generation of hierarchical micro-mesoporous-macroporous structure can facilitate electron-hole transportation, the adsorption of reactants and the desorption of the products; (iv) the agglomeration of catalytic AuPt particles can be suppressed via the protection of the outer shell. Our synthetic strategy is a unique approach for the rational design of intricate hollow-structured photocatalysts.

## RESULTS AND DISCUSSION

**Figure 1.**
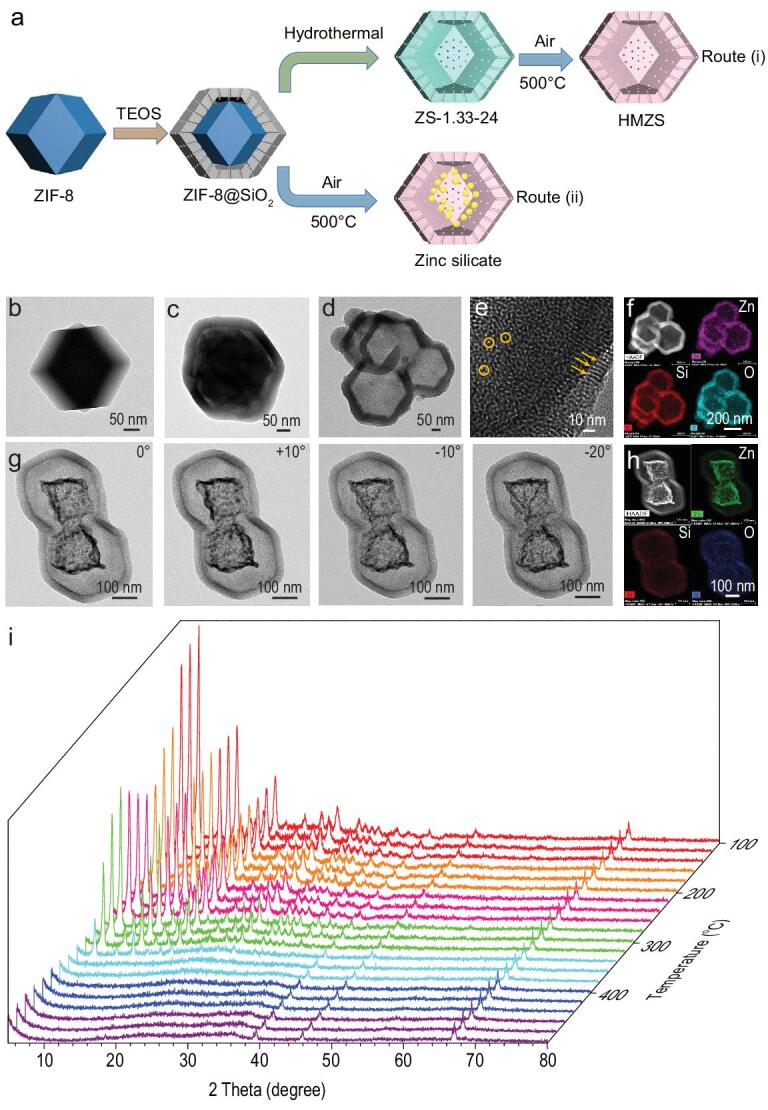
(a) Schematics illustration of the hollow-structured silica composites. Blue, grey, green and pink colors refer to ZIF-8, SiO_2_ layer, hollow zinc silica composites with CTAB and hollow zinc silica composites, respectively. TEM images of ZIF-8 (b), ZIF-8@silica before hydrothermal (c) and HMZS (d, e), respectively. (f) HAADF image and element mapping images of HMZS. (g) TEM images with the sample holder tilted to 0°, +10°, −10° and −20° by rotation around the axis of the holder. (h) HAADF image and element mapping images of zinc silicate. (i) *In situ* XRD patterns of the ZIF-8@SiO_2_ with increasing calcination temperature under air atmosphere.

The synthesis strategy of hollow-structured zinc silica composites (ZS) is schematically depicted in Fig. 1a. In the first step, the Stöber coating method is used to deposit a silica layer on the surface of ZIF-8. After generating highly uniform ZIF-8@SiO_2_ core-shell structures, hydrothermal treatment was used to prepare the hollow-structured silica composites. The hydrothermal-treated ZIF-8@SiO_2_ particles are denoted ZS-n-x, where n and x represent the molar amount of tetraethyl orthosilicate (TEOS) and hydrothermal treatment time. To remove hexadecyltrimethylammonium bromide (CTAB), hollow-structured silica composites were calcined in air to achieve HMZS particles. The as-obtained HMZS with different shell thickness are denoted HMZS-n, where n represent the molar amount of TEOS. To prepare hollow-structured Au/Pt photocatalysts, a wet-chemical reduction method was used to load Au or Pt nanoparticles within the hollow particles or on the surface of the hollow particles by reduction of Au or Pt salts.

The X-ray diffraction (XRD) patterns of ZIF-8 crystals (Fig. S1) agree well with previous reports [20,21] and the transmission electron microscopy (TEM) image of ZIF-8 particles (Fig. 1b) shows a polyhedral structure with a smooth surface and average particle size of ∼300 nm. After coating the SiO_2_ layer to form a ZIF-8@SiO_2_ core-shell structure, the particles retain their polyhedral shape but exhibit a rougher surface, as shown in Fig. 1c. Figure 1d and e shows that hollow-structured HMZS nanoparticles with shell thicknesses of ∼50 nm and pore size of ∼3 nm are generated after hydrothermal treatment for 24 h followed by calcination in the air. The high-angle annular dark-field scanning transmission electron microscopy (HAADF-STEM) image and the corresponding energy dispersive X-ray spectroscopy elemental mapping images (Fig. 1f) were used to find that elemental zinc, silica and oxygen are homogenously distributed in the whole framework. The XRD patterns of HMZS were shown in Fig. S2a and only one broad peak at 25° was detected, which can be attributed to amorphous SiO_2_. The porosity of the HMZS composites was then studied by nitrogen adsorption–desorption analysis. The HMZS composites possess a mixed type I and IV isotherms (Fig. S2b). A hierarchical porous structure with pore size centered at 1.4 nm and 2.7 nm based on Density Functional Theory (DFT) calculation is observed (inset in Fig. S2b). The Brunauer–Emmett–Teller (BET) surface area and the pore volume of HMZS are 842 m^2^ g^−1^ and 0.54 cm^3^ g^−1^, respectively. As shown in Fig. S3 and Table S1, when using less TEOS concentration of 0.67 mol, the obtained HMZS-0.67 particles exhibit lower BET surface area and a small portion of micropores. After increasing the TEOS concentration to 2 mol, it is clear that the surface area has also enlarged with a higher portion of micropores and similar pore size distribution. The relatively lower BET surface area of HMZS-0.67 composite seems to suggest increasing interactions between zinc particles and silica matrix.

Additionally, through direct calcination of ZIF-8@SiO_2_ at 500°C, yolk-shell structured particles can be achieved (Fig. 1g and h) and a phase transformation has occurred at 360°C through the *in situ* XRD examinations (Fig. 1i) during the calcination process. The peaks at 39.5°, 46.1° and 67.3° for the ZIF-8@SiO_2_ particles calcined at 360°C can be ascribed to zinc silicate (JCPDS 23–1172). The peaks around 24° can be attributed to amorphous SiO_2_.

**Figure 2.**
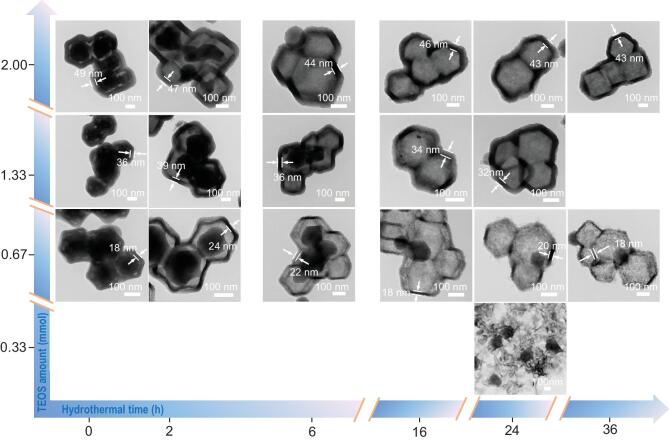
TEM images of ZIF-8@SiO_2_ with different TEOS amounts and hydrothermal time.

The time evolution of this specific hollow structure through hydrothermal treatment of ZIF-8@SiO_2_ was studied to understand its formation mechanism. Based on the TEM results (Fig. 2) and the relationship between the size of ZIF-8 and the amount of TEOS (Fig. S4), the total disappearing times of ZIF-8 particles for HMZS-2 and HMZS-1.33 are ∼6 h and ∼16 h, while ZIF-8 particles are still present within the hydrothermal treatment of 36 h. It can be concluded that a thicker SiO_2_ shell derived from extra addition of TEOS resulted in the faster transformation from core-shell structure to hollow structure. As shown in the TEM images of HMZS-0.67–2 samples in Fig. 2, it can be clearly observed that two layers formed on the surface for HMZS-0.67–2 at the hydrothermal time for 2 h. This indicates the adhesion forces between the outer layer of ZIF-8 particles and the SiO_2_ layer. With an increase in hydrothermal times, the double layers are merged into one layer. Therefore, the pores within the particles and on the surface can be blocked by the infiltrated zinc particles, leading to the relatively smaller surface area and pore volume.

The composite XRD pattern (Figs S5–S8) at different hydrothermal times and various SiO_2_ content highlights the formation of hollow-structured zinc silica composites. The characteristic peaks of ZIF-8 particles in the XRD patterns disappeared more quickly as TEOS amount increased, which is consistent with the TEM results in Fig. 2. In addition, irregularly shaped particles with decreased average particle size of about 110 nm are only produced without coating on the surface of ZIF-8 [13]. This indicates that the SiO_2_ coating is essential for the formation of hollow structure and the evolution of ZIF-8. Herein, we propose an ‘adhesive-contraction’ formation mechanism of hollow structure through hydrothermal treatment of ZIF-8@SiO_2_ core-shell structures, which have some similarities with the formation mechanism of hollow microspheres derived from thermal decomposition of solid precursors [22]. At the initial stage of hydrothermal treatment, there is a non-equilibrium heat treatment between the surface of ZIF-8 particles and the inside of ZIF-8 particles. As the hydrothermal treatment continues, two forces in opposite directions are supposed to act on the interface between the SiO_2_ shell and ZIF-8 core due to the gradual decomposition of ZIF-8. The contraction force caused by the oxygenolysis of the organic species leads to the inward contraction of ZIF-8 core, meanwhile, the adhesive force between ZIF-8 particles and the shell of SiO_2_ prevents the inward contraction of ZIF-8 particles. With increasing hydrothermal time, the SiO_2_ layer can pull the surface of ZIF-8 particles outward under the intense adhesion force between ZIF-8 particles and the shell of SiO_2_, leading to the self-separation of ZIF-8 particles. The remaining interior ZIF-8 cores can be decomposed by prolonging hydrothermal time and the large voids within the shells are formed. Finally, the ZIF-8 cores can fully disappear, leading to the generation of a hollow structure.

**Figure 3.**
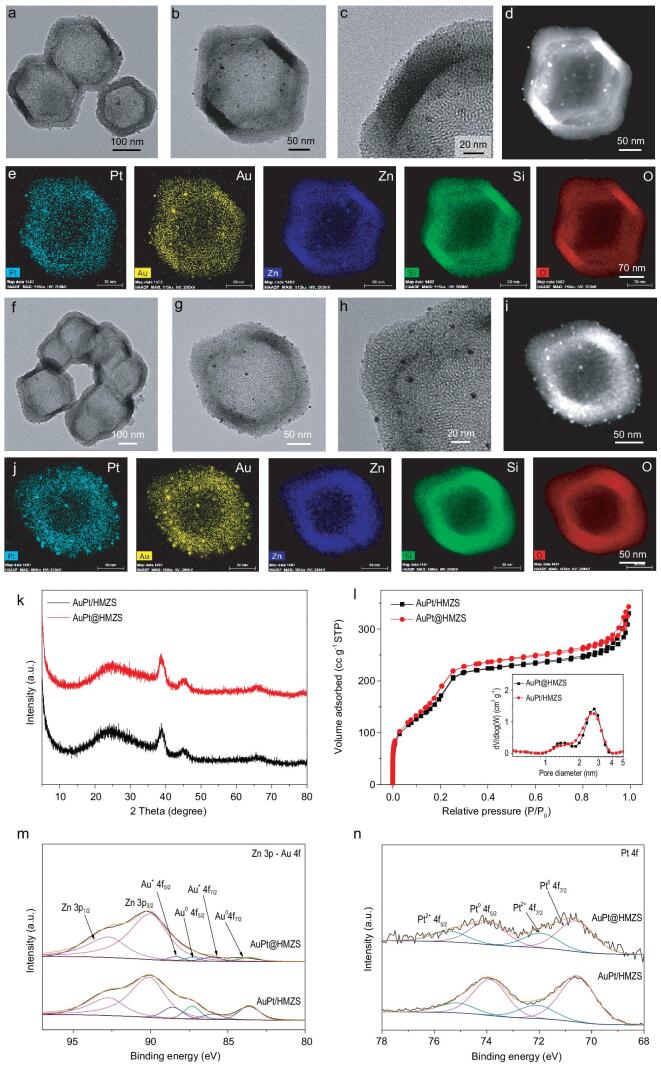
(a–c) TEM images, (d) HAADF image and (e) element mapping images of AuPt@HMZS. (f–h) TEM images, (i) HAADF image and (j) element mapping images of AuPt/HMZS. (k) XRD pattern, (l) N_2_ adsorption/desorption isotherms and pore size distribution curves (inset), (m) XPS spectra of Zn 3p-Au 4f and (n) Pt 4f of AuPt@HMZS and AuPt/HMZS.

The as-prepared hollow structure HMZS particles were used to selectively deposit monometallic nanoparticles and bimetallic nanoparticles (Au and Pt) within the inner shell or on the outer shell of the hollow structure. TEM images (Fig. 3a–d, Figs S9 and S10) show Au, Pt and bimetallic nanoparticles are successfully confined within the hollow structure. To determine the dispersion of elements in Au@HMZS, Pt@HMZS and AuPt@HMZS composites, HAADF-STEM imaging and elemental mapping (Fig. 3e, Figs S9 and S10) were used to reveal that silica, zinc and oxygen atoms are homogeneously distributed in the whole framework, and metal nanoparticles (Au and Pt) are also situated within silica shells. For AuPt/HMZS materials, the TEM images (Fig. 3f–i), HAADF-STEM imaging and elemental mapping (Fig. 3j) show Au, Pt and bimetallic nanoparticles are successfully loaded on the surface of the hollow structure through the impregnation method. The XRD pattern of Au@HMZS in Fig. S11 exhibited characteristic peaks of Au (JCPDS 04–0784), while undetectable Pt peaks can be found in the XRD pattern of Pt@HMZS due to the possible formation of small Pt nanoparticles. For AuPt@HMZS and AuPt/HMZS, broad peaks at 38.9°, 45.2° and 66.1° were observed (Fig. 3k), indicating the formation of bimetallic Au/Pt nanoparticles. Based on the Scherrer equation, the crystalline size of metal nanoparticles in Au/HMZS, AuPt/HMZS and AuPt@HMZS catalysts are 11 nm, 2.7 nm and 3.1 nm respectively. Nitrogen adsorption isotherms of four samples in Fig. 3l and Fig. S12a show a combination of types I and IV isotherms, indicating the presence of micropores and mesopores. This is also confirmed from the pore size distribution in the inset of Fig. 3l and Fig. S12b with a hierarchical size centered at 1.4 nm and 2.7 nm. The BET surface area and the pore volume are 802 m^2^ g^−1^ and 0.53 cm^3^ g^−1^ (Table 1) for AuPt@HMZS, which are a little higher than that of AuPt/HMZS (732 m^2^ g^−1^ and 0.51 cm^3^ g^−1^). Inductively coupled plasma optical emission spectrometry (ICP-OES) analysis of AuPt@HMZS indicates that the Au and Pt loading (mass%) was determined to be approximately 0.8% and 0.7% respectively. As shown in Table 1, a similar Au and Pt loading can be achieved for AuPt@HMZS materials. Figure S13 illustrates the X-ray photoelectron spectroscopy (XPS) survey spectra of AuPt@HMZS, confirming the existence of carbon (9.72 at%), silica (30.69 at%), oxygen (55.84 at%), zinc (3.73 at%), Pt (0.01 at%) and Au (0.01 at%). Compared with the negligible amount of metal particles from the surface of AuPt@HMZS, a relatively higher atomic ratio of metal particles and higher intensities are observed from the XPS survey spectra of AuPt/HMZS (Table S2), indicating the successful impregnation on the surface of HMZS. After deconvolution of Au 4f peaks (Fig. 3m), the peaks at the binding energies of 83.6 and 87.3 eV are assigned to Au^0^ 4f 7/2 and Au^0^ 4f 5/2 electrons of Au metal, respectively. The peaks at 86.0 and 88.4 eV correspond to the binding energies of the 4f 7/2 and 4f 5/2 orbitals of Au^+^ species, respectively. Two asymmetric peaks of the Zn 3p spectra at 90.0 and 92.7 eV (Fig. 3m) overlying Au chemical state were assigned to Zn^2+^ 3p 3/2 and Zn^2+^ 3p 1/2. For the XPS spectrum of Pt 4f spectra in Fig. 3n, the peak at the binding energies of 70.6 and 74.0 eV are assigned to the 4f 7/2 and 4f 5/2 of Pt metal, while the Pt^2+^ 4f 7/2 and 4f 5/2 appear at 72.0 and 75.4 eV [23–25]. The binding energies of AuPt/HMZS catalysts are similar with those of AuPt@HMZS, indicating both catalysts have similar oxidation states and alloying natures. However, the peak intensities of Au and Pt 4f peak become obvious compared with those of AuPt@HMZS, confirming the loading of Au and Pt particles on the surface of HMZS.

**Table 1.** Physical properties of zinc silica composites.

Catalysts	S_BET_^a^ (m^2^ g^−1^)	S_micro_^b^ (m^2^ g^−1^)	S_micro_/S_meso_^b^	Pore size distribution^c^ (nm)	V_t_^d^ (cm^3^ g^−1^)	V_micro_^e^ (m^3^ g^−1^)	V_micro_/V_meso_^f^	Metal crystalline size^g^ (nm)	Metal content^h^ (%)

AuPt/HMZS	732	665	10	1.4, 2.7	0.51	0.31	2.6	2.7	Au (0.7), Pt (0.7)
AuPt@HMZS	802	732	11	1.4, 2.7	0.53	0.33	1.6	3.1	Au (0.8), Pt (0.7)

^a^S_BET_ is the Brunauer-Emmett-Teller (BET) specific surface area. ^b^S_micro_ is the t-plot-specific micropore surface area calculated from the N_2_ adsorption-desorption isotherm. ^b^S_meso_ is the specific mesopore surface area estimated by subtracting S_micro_ from S_BET_. ^c^Pore size distribution is obtained by DFT method. ^d^V_t_ is the total specific pore volume determined by the adsorption branch of the N_2_ isothermat P/P_0_ = 0.99. ^e^V_micro_ is the t-plot-specific micropore volume calculated from the N_2_ adsorption-desorption isotherm. ^f^V_meso_ is the specific micropore volume calculated by subtracting V_micro_ from V_t_. ^g^Calculated by the Scherrer equation in the XRD profiles. ^h^Metal content was determined through ICP-OES analysis.

**Figure 4.**
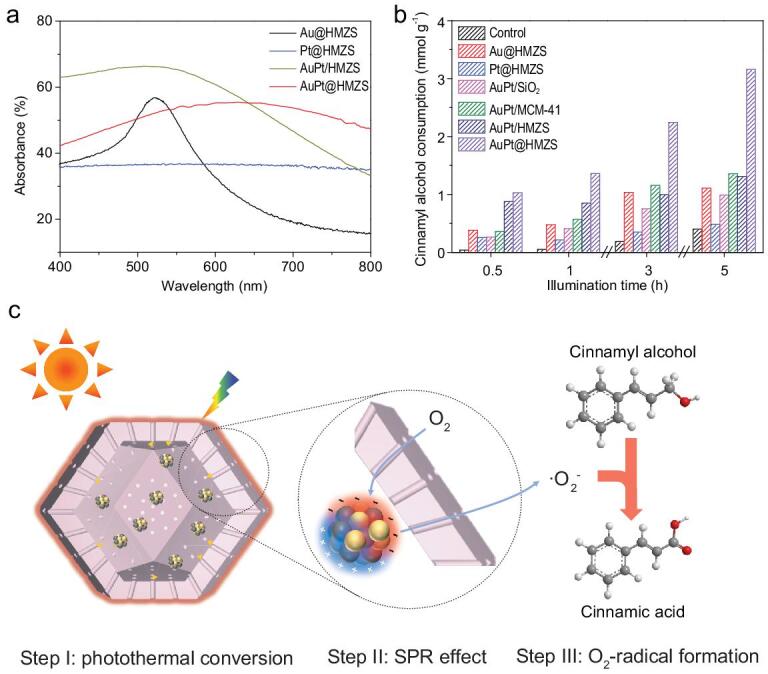
(a) UV-Vis spectra of Au@HMZS, Pt@HMZS, AuPt/HMZS and AuPt@HMZS. (b) Comparison of photocatalytic oxidation performance from cinnamyl alcohol solution by using Au@HMZS, Pt@HMZS, AuPt/SiO_2_, AuPt/MCM-41, AuPt/HMZS, AuPt@HMZS and control experiment (without irradiation). (c) Proposed mechanism responsible for the photocatalytic oxidation of cinnamyl alcohol in the presence of AuPt@HMZS.

The UV-Vis absorption spectra for Au@HMZS, Pt@HMZS, AuPt/HMZS and AuPt@HMZS are shown in Fig. 4a. The as-prepared catalysts Pt@HMZS showed a broad absorption band at λ > 400 nm due to the inter-band transition of Pt nanoparticles on the surface [26]. The spectrum of Au@HMZS presented a broad absorption band in the visible light absorption region with the peak centering at about 520 nm which could be attributed to the SPR effect of Au nanoparticles on the surface [27]. Compared with Au@HMZS, AuPt@HMZS and AuPt/HMZS, catalysts exhibited broader absorbance region under visible light and the adsorption edge displayed a red-shift, indicating the influence of strong metal–metal interactions at the alloy interface [28]. Additionally, the differences between the spectra of bimetallic nanoparticles and the monometallic ones further confirm the formation of Au−Pt alloy composites [29]. A similar band adsorption by using Au/SiO_2_, Au/MCM-41, Pt/SiO_2_, Pt/MCM-41, AuPt/SiO_2_ and AuPt/MCM-41 was observed (Fig. S14).

To evaluate the photocatalytic performance of the as-prepared hollow catalysts, the photocatalytic oxidation of cinnamyl alcohol was performed under visible light irritation. Photocatalytic oxidation activities for cinnamyl alcohol solution are provided in Fig. 4b. Included in the figure are activities for Au@HMZS, Pt@HMZS, AuPt@HMZS, AuPt/HMZS and control experiments (without irradiation). The control experiments show that traceable cinnamic acid is generated in the absence of irradiation. Cinnamyl alcohol was converted into cinnamic acid with a high selectivity of >99% for all of the samples. The cinnamyl alcohol consumption for monometallic catalyst Pt@HMZS is ∼0.5 mmol g^−1^ after 5 h of visible light irradiation, while 1.1 mmol g^−1^ of cinnamyl alcohol was converted when Au@HMZS catalyst was used. This shows that the existence of Au metal with SPR-induced effect facilitates charge injection from Au metal to hollow structure and enables the reduction of O_2_ molecular to superoxide radicals (•O_2_^−^) [30]. The catalytic activity of the AuPt@HMZS catalysts was 3.1 mmol g^−1^ after 5 h irradiation, which is significantly higher than that of the monometallic catalysts Au@HMZS (six folds higher) and Pt@HMZS. This improvement can be attributed to the synergistic effect of the electron-trapping function of Pt metal and SPR effect of Au metal vital for the charge separation and catalytic oxidation reaction [31]. In addition, the photocatalytic oxidation activity of AuPt@HMZS catalysts is superior to those of bimetallic AuPt/SiO_2_ and AuPt/MCM-41 catalysts with similar amounts of Au and Pt content, monometalic Au/SiO_2_ (Fig. S15), Pt/SiO_2_ (Fig. S15), Au/MCM-41 (Fig. S16) and Pt/MCM-41 (Fig. S16) catalysts. To further highlight the superior photocatalytic performances of AuPt@HMZS catalysts, AuPt/HMZS catalysts with the bimetallic metal nanoparticle on the surface of HMZS were also investigated for the photocatalytic oxidation of cinnamyl alcohol. Compared to AuPt/HMZS catalysts with similar AuPt loading, particle size, metal dispersion and surface area, the conversion of cinnamyl alcohol by using AuPt@HMZS was also higher. This can be attributed to the fact that the multi-scattering of light can be enhanced within the hollow structure when bimetallic nanoparticles were confined within the hollow structure, further strengthening the SPR effect and photocatalytic activity [32,33]. As listed in Table S3, the as-synthesized AuPt@HMZS catalysts shows superior photo-oxidation performance compared to other reported studies.

Based on the above-mentioned results, a proposed mechanism for photocatalytic oxidation of cinnamyl alcohol in the presence of AuPt@HMZS is presented in Fig. 4c. Firstly, due to the SPR effect derived from the collective oscillation of surface electrons, the generated electrons migrated to the surface of Au species and were injected into the Pt species. Subsequently, the dissolved O_2_ molecules in the aqueous solution could react with the generated electron from the Pt species to produce •O_2_^−^ radicals [34]. The formed •O_2_^−^ radicals are responsible for the catalytic oxidation of the reactant. Meanwhile, under illumination, heat can be generated through a photothermal conversion process. Multi-scattering of light can occur with the light absorption within the hollow structure, enhancing light-harvesting ability and elevating the inside temperature of the catalysts. The increased reaction temperature inside the nanoreactor will further improve the reaction rate. The electron paramagnetic resonance (EPR) was used to probe the production of reactive oxygen species. The recorded EPR data have been displayed in Fig. S17, where the characteristic peaks of superoxide radicals were apparently observed in the reaction system, which is in agreement with previous studies [35,36]. To further confirm the formation of superoxide radicals, methanol was then added to the reaction solution to quench hydroxyl radicals (•OH^−^). It was observed that the signal intensities are still obvious, indicating that superoxide radicals are the dominant generated radicals. These as-formed radicals are responsible for the deprotonation of cinnamyl alcohol. In addition, due to the SPR-induced effect from Au metal, the accumulated hot electrons generated on the sites of Au metal can transfer to Pt metal because Pt metal possesses larger work function than Au metal. The SPR effect of Au metal in the AuPt@HMZS particles can not only provide more electrons for the photocatalytic oxidation reaction, but also decrease the probability of charge recombination.

## CONCLUSION

In summary, we have demonstrated a facile synthesis of hollow-structured photocatalysts with controllable spatial location of active metals, chemical compositions and tunable shell thickness. Hollow structures can be achieved through coating SiO_2_ on the surface of ZIF-8 and a subsequent hydrothermal treatment. The formation mechanism of hollow structure has been systematically investigated and an ‘adhesive-contraction’ model has been proposed. The hollow-structured AuPt@HMZS photocatalysts exhibited a synergetic effect among plasmonic hot electron injection and electron trapping, improving solar energy utilization and electron-hole separation of photocatalysts. This work is significant to the development of novel and efficient hollow-structured photocatalysts.

## Supplementary Material

nwaa080_Supplemental_File
