# Supplementary material for: Construction of hollow mesoporous silica nanoreactors for enhanced photo-oxidations over Au-Pt catalysts
Source: Natl Sci Rev. 2020 Apr 24;7(11):1647–55. doi: 10.1093/nsr/nwaa080 (PMC8288811; doi:10.1093/nsr/nwaa080)
Supplement: nwaa080_Supplemental_File [file nwaa080_supplemental_file.docx]

Supporting Information

Construction of Smart Hollow Mesoporous Silica Nanoreactors for Enhanced Photo-oxidations over Coupled Au-Pt Catalysts

*Hao Tian,^a,b^ Jinhui Zhao,^c^ Xinyao Wang,^a^ Lizhuo Wang,^c^ Hao Liu,^b^ Guoxiu Wang,^b^ Jun Huang,^c^* Jian Liu^a,d*^ and G. Q. (Max) Lu^e^*

^a^ State Key Laboratory of Catalysis, Dalian Institute of Chemical Physics, Chinese Academy of Sciences, Dalian 116023, China

^b^ Centre for Clean Energy Technology, School of Mathematical and Physical Sciences, Faculty of Science, University of Technology Sydney, Sydney, NSW 2007, Australia

^c^ Laboratory for Catalysis Engineering, School of Chemical and Biomolecular Engineering, Sydney Nano Institute, The University of Sydney, Sydney, NSW 2006, Australia

^d^ DICP-Surrey Joint Centre for Future Materials, Department of Chemical and Process Engineering and Advanced Technology Institute, University of Surrey, Guildford, GU2 7XH, UK

^e^ University of Surrey, Guildford, Surrey GU2 7XH, UK

Dedicated to the scientific work of Prof. Can Li on the occasion of his 60th birthday

***Corresponding author.** Emails:

[jianliu@dicp.ac.cn](mailto:jianliu@dicp.ac.cn); [jian.liu@surrey.ac.uk](mailto:jian.liu@surrey.ac.uk); [jun.huang@sydney.edu.au](mailto:jun.huang@sydney.edu.au)

1. **Experimental Section**

**1.1 Chemicals and reagents**

The chemicals used in this work such as hexadecyltrimethylammonium bromide (CTAB), tetraethyl orthosilicate (TEOS), ammonia solution (25%), ethanol (95-100%), methanol (99%), zinc nitrate hexahydrate (Zn(NO_3_)_2_·6H_2_O), gold chloride trihydrate (HAuCl_4_·3H_2_O), chloroplatinic acid hexahydrate (H_2_PtCl_6_·6H_2_O), 2-methylimidazole and hexadecyltrimethylammoniumchloride solution (CTACl, purum, 25% in H_2_O) were purchased from Sigma-Aldrich and used as received without any further purification. Washing was achieved with ultrapure water and reagent grade ethanol where required. Ultrapure water was used for solution preparations.

**1.2 Synthesis of ZIF-8**

Typically, Zn(NO_3_)_2_·6H_2_O (0.89 g) was dissolved in 30 mL of methanol to form a solution. 20 mL of methanol containing 2-methylimidazole (1.97g) was poured into Zn(NO_3_)_2_ solution. The mixture was kept stirring at room temperature for 24 h. The resulting white precipitates was collected by centrifugation, washed with methanol three times, and finally dried at 100 °C overnight.

**1.3 Synthesis of Au/ZIF-8, Pt/ZIF-8**

As-synthesized ZIF-8 crystals (0.1 g) were immersed in a solution of HAuCl_4_·3H_2_O (5.2 mg) in methanol (1.0 mL) and the mixture was sonicated for 5 minutes. After the mixture was dried at 100°C for 0.5 hours, methanol (10 mL) was added. The mixture was sonicated for 5 minutes and then a solution of NaBH_4_ (40 mg) in methanol (1.0 mL) was added quickly to the mixture under strong stirring. The reaction was allowed to proceed for another 1.5 h. The products Au/ZIF-8 were collected by centrifugation, washed with methanol several times, and dried at 100 °C overnight. The synthesis procedure of Pt/ZIF-8 is similar with that of Au/ZIF-8 through simply changing HAuCl_4_∙3H_2_O to H_2_PtCl_6_∙6H_2_O with the same addition amount.

**1.4 Synthesis of AuPt/ZIF-8**

Dried ZIF-8 crystals (0.1 g) were immersed in a solution of HAuCl_4_·3H_2_O (2.6 mg) and H_2_PtCl_6_∙6H_2_O (2.6 mg) in methanol (1.0 mL) and the mixture was sonicated for 5 minutes. After the mixture was dried at 100°C for 0.5 hours, methanol (10 mL) was added. The mixture was sonicated for 5 minutes and then a solution of NaBH_4_ (40 mg) in methanol (1.0 mL) was added quickly to the mixture under strong stirring. The reaction was allowed to proceed for another 1.5 h. The products Au/ZIF-8 were collected by centrifugation, washed with methanol several times, and dried at 100 °C overnight.

**1.5 Synthesis of HMZS, Au@HMZS, Pt@HMZS, AuPt@HMZS**

In a typical synthesis, CTAB (0.1 g) was dissolved in a mixture of water (20 mL) and ethanol (8 mL). Then, an aqueous solution of ammonia (NH_4_OH, 0.2 mL, 25 wt%) was added and stirred at room temperature for 0.5 h, followed by addition of 0.1g ZIF-8. After stirring for 0.5 h, 300 μL TEOS solution was added into that suspension. The mixture was stirred for 24 h at room temperature and subsequently heated for 24 h at 100 °C under static conditions in a Teflon-lined autoclave. After centrifugation and dried at 100 °C for 24 h, the obtained solid products were calcined in a muffle furnace using a heating rate of 2 °C min^-1^ up to 500 °C, dwell for 4 h to obtain the HMZS particles. The synthesis procedure of Au@HMZS, Pt@HMZS, AuPt@HMZS is similar with that of HMZS through simply changing ZIF-8 to Au/ZIF-8, Pt/ZIF-8, AuPt/ZIF-8 with the same addition amount.

**1.6 Synthesis of** **AuPt/HMZS**

The obtained products HMZS were immersed in a solution of HAuCl_4_·3H_2_O (5.2 mg) and H_2_PtCl_6_∙6H_2_O (5.2 mg) in methanol (1.0 mL) and the mixture was sonicated for 5 minutes. After the mixture was dried at 100°C for 0.5 hours, methanol (10 mL) was added. The mixture was sonicated for 5 minutes and then a solution of NaBH_4_ (40 mg) in methanol (1.0 mL) was added quickly to the mixture under strong stirring. The reaction was allowed to proceed for another 1.5 h. After centrifugation, wash with methanol several times, and dried at 100 °C overnight, the obtained solid products were calcined in a muffle furnace using a heating rate of 2 °C min^-1^ up to 500 °C, dwell for 4 h to obtain the AuPt/HMZS.

**1.7 Synthesis of Pt/SiO_2_ and Au/SiO_2_**

The Pt@SiO_2_ and Au@SiO_2_ catalysts were prepared by the impregnation method. 200 mg SiO_2_ (silica, fumed, Aldrich) was mixed with metallic salt aqueous solution (H_2_PtCl_6_ (Hydrogen hexachloro-platinate(IV) solution, Sigma-Aldrich, 8% in water), HAuCl_4_ (Gold(III) chloride trihydrate, Sigma-Aldrich, 99.9% trace metals basis)), stirring under magnetic stirrer for 24 h. The suspension was dried in oven at 80 °C overnight and calcined at 550 °C for 4 hours. The calculated metal loading rate is 1.5 %.

**1.8 Synthesis of AuPt/SiO_2_**

The AuPt@ SiO_2_ catalysts were prepared by the impregnation method. 200 mg SiO_2_ (silica, fumed, Aldrich) was mixed with platinum salt aqueous solution (H_2_PtCl_6_) and gold salt aqueous solution (HAuCl_4_), stirring under magnetic stirrer for 24 h. The suspension was dried in oven at 80 °C overnight and calcined at 550 °C for 4 hours. The calculated metal loading rate is 1.5 %.

**1.9 Synthesis of MCM-41**

The CTACl solution was mixed with ammonia solution (25%) and TEOS in a volume ratio of 1:1:1 in demineralized water (500 mL) and stirred at room temperature to form a white gel. The resulting solids were collected by filtration, washed with distilled water, and then dried in an oven at 80 °C. Finally, the obtained MCM-41 materials were calcined at 550 °C with a heating rate of 1 °C/min in the presence of static air for 6 h.

- 1. **Synthesis of** **Au/MCM-41, Pt/MCM-41 and AuPt/MCM-41**

The Au/MCM-41, Pt/MCM-41 and AuPt/MCM-41 catalysts were synthesized with the similar preparation method with Au/SiO_2_, Pt/SiO_2_ and AuPt/SiO_2_ through replacing SiO_2_ with MCM-41.

**1.11 Characterization**

The sample morphology was characterized by using a transmission electron microscope (TEM, JEOL EM-2100). High angle annular dark field scanning transmission electron microscopy (HAADF-STEM) imaging and elemental mapping were carried out using a FEI Titan G2 80-200 TEM/STEM with ChemiSTEM Technology operating at 200 kV. The elemental maps were obtained by energy dispersive X-ray spectroscopy using the Super-X detector on the Titan with a probe size ~1 nm and a probe current of ~0.4 nA. Powder X-ray powder diffraction (XRD) analysis was performed on an X-ray diffractometer (Bruker D8 Advance) using Cu Kα radiation at 40 kV and 30 mA. The BET specific surface area and single-point pore volume were obtained from nitrogen adsorption isotherms measured at -196 °C using a nitrogen sorption instrument (ASAP 2460 Micropore Physisorption Analyzer). Prior to nitrogen adsorption measurements, the samples were degassed at 250 °C overnight. Metal contents of photocatalysts were determined by Inductively Coupled Plasma Optical Emission Spectrometry (ICP-OES). X-ray photoelectron spectroscopy (XPS) was collected on a VG ESCALAB MK2 apparatus by using Al Ka (hl ¼ 1486.6 eV) as the excitation light source. The Electron paramagnetic resonance (EPR) was conducted on a Bruker EMS-plus instrument to record the evolution of free radicals and the data were analyzed by Xeon software. The operating conditions are: centre field, 3514.7; sweep width, 100 G; scan number, 3; microwave attenuation, 10 dB. 5,5-dimethyl-1-pyrroline N-oxide (DMPO) was used as spin-trapping agent.

**1.12 Oxidation of** **Cinnamyl alcohol**

The oxidation reaction was carried out in a photocatalysis autoclave (micro reactor, Yanzheng Instrument) with a magnetic stirring under visible light irritation (Lamp house R400-3JH, with a UV-cut filter, >420 nm). The autoclave was filled with reaction mixture (10 mL 0.01 M cinnamyl alcohol (water solution)) and 10 mg catalysts and was purged with 2 bar O_2_ as the oxidant. The reaction products were analysis by Shimazu-2014 gas chromatography (GC) equipped with a flame ionization detector (FID) and Rtx-wax column.


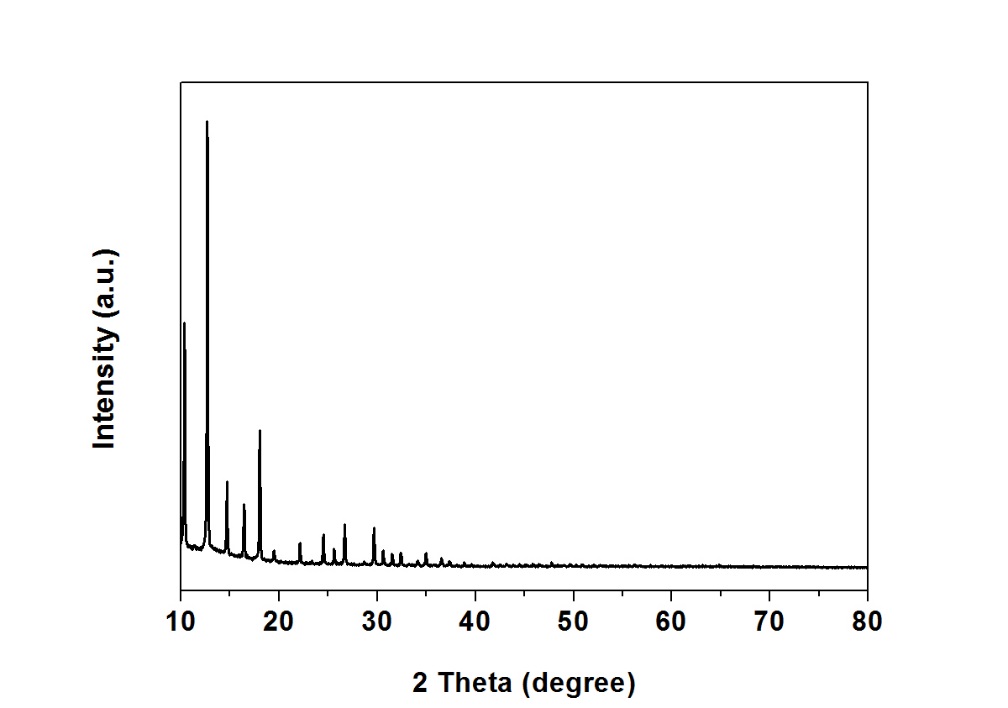


**Figure S1.** XRD pattern of ZIF-8.


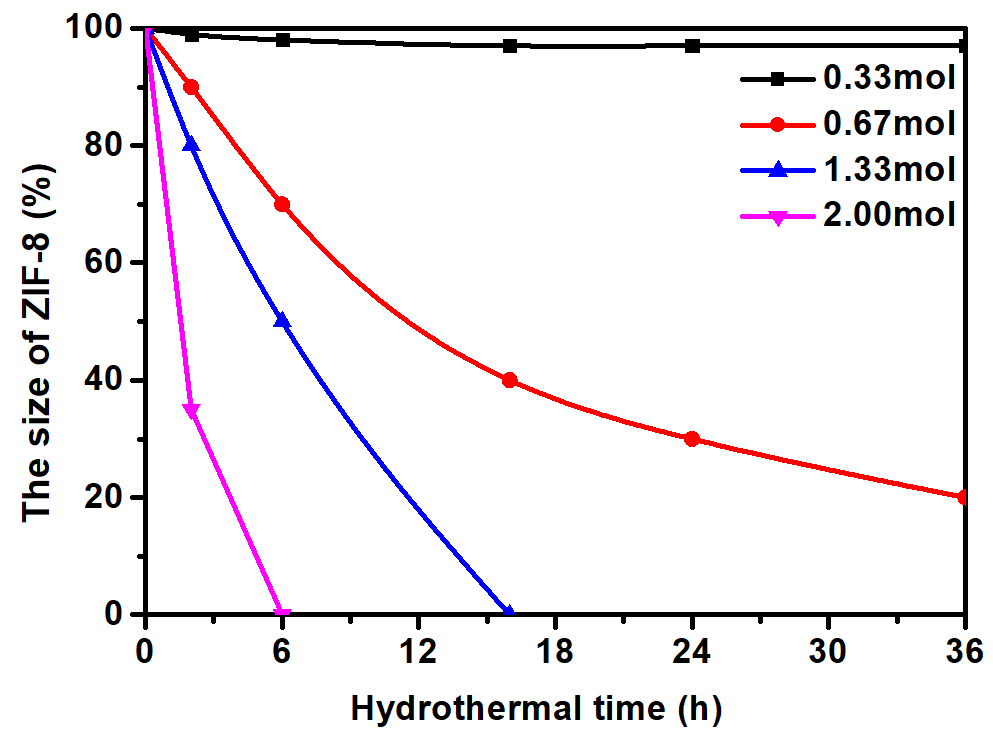


**Figure S2.** The relationship between the size of ZIF-8 and the hydrothermal time.

**
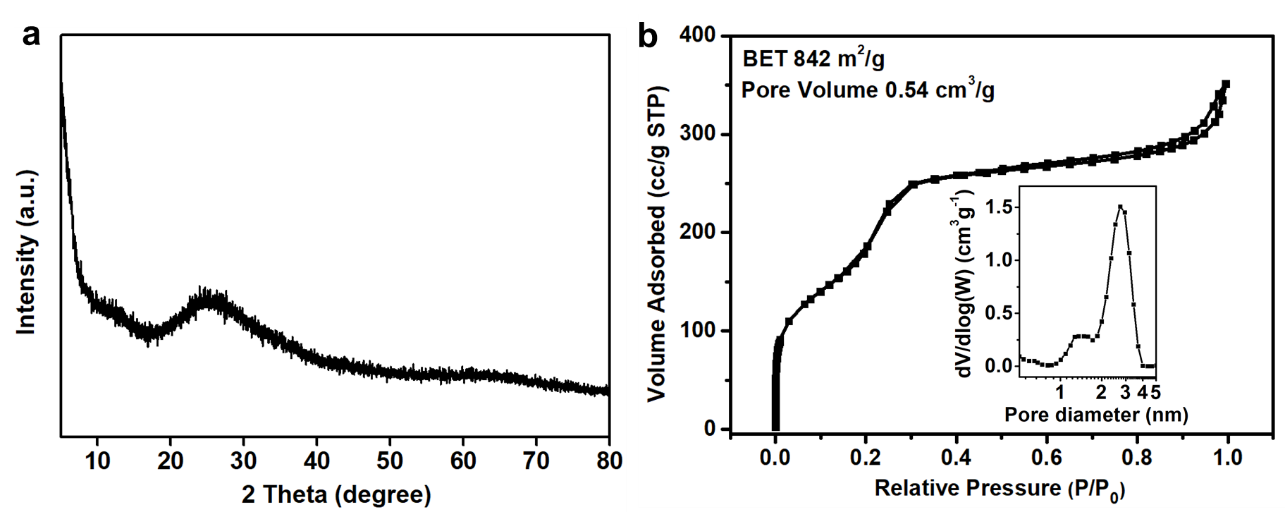
**

**Figure S3.** (a) XRD pattern and (b) N_2_ adsorption–desorption isotherm and pore size distribution curves (inset) of HMZS.


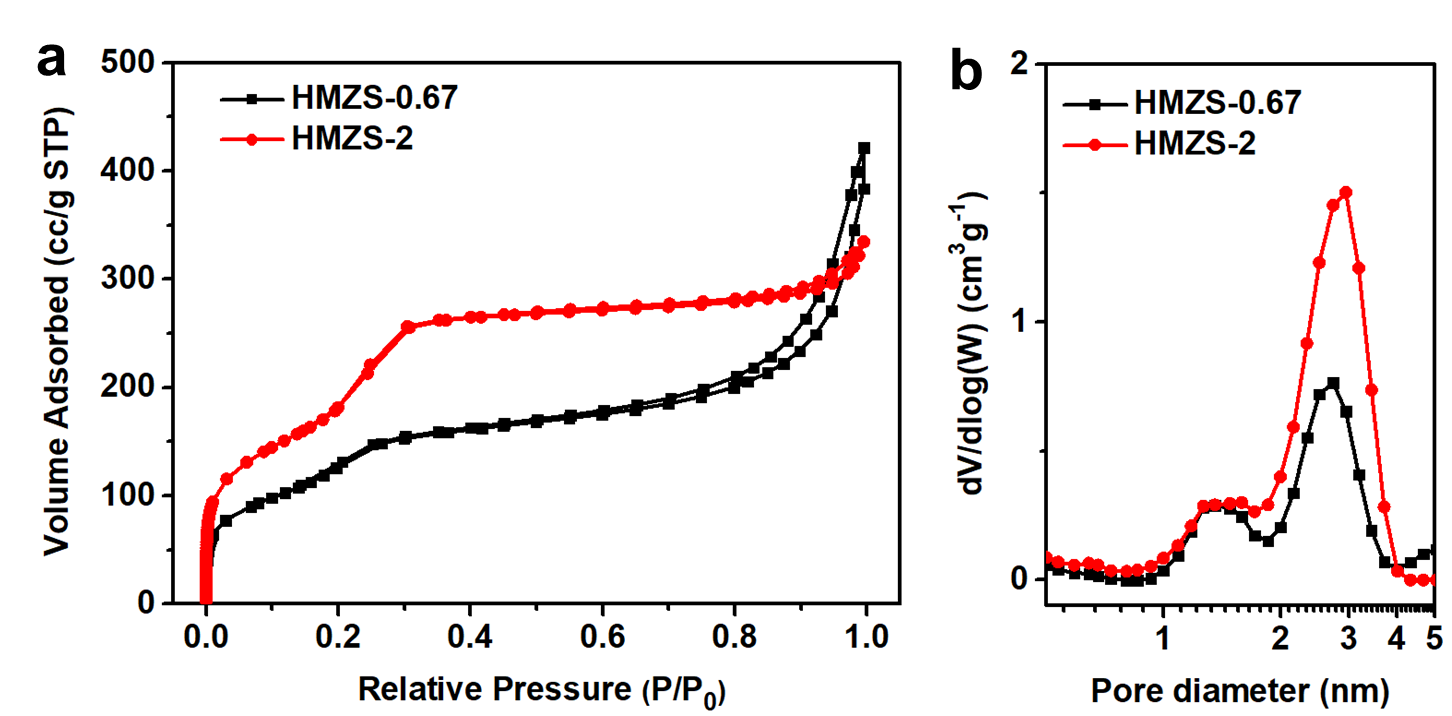


**Figure S4.** N_2_ sorption isotherms (a) and pore size distribution curves (b) of HMZS-0.67 and HMZS-2.

**
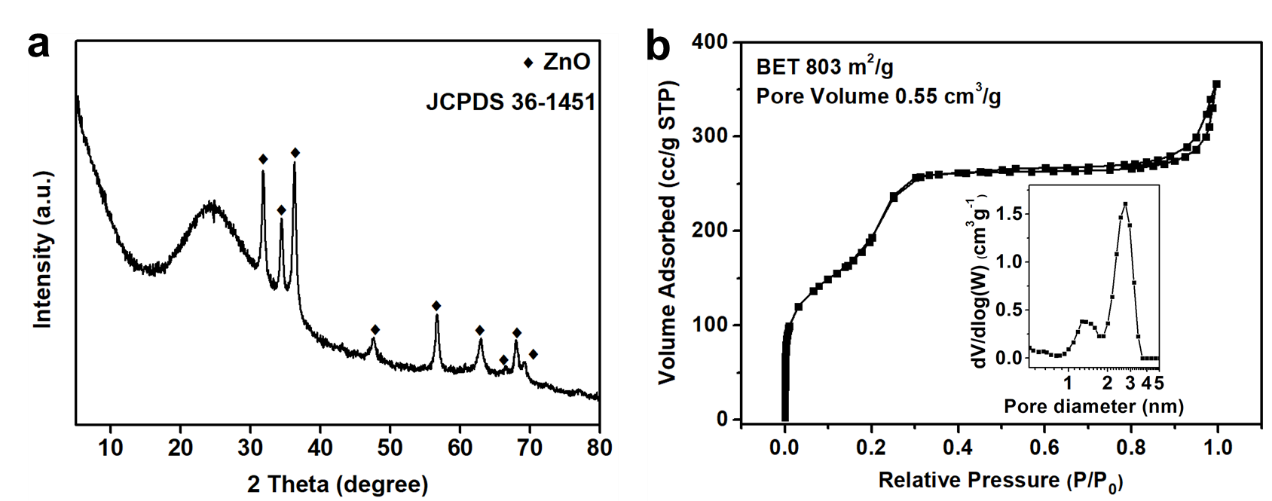
**

**Figure S5**. (a) XRD pattern and (b) N_2_ adsorption–desorption isotherm pore size distribution curves (inset) of ZnO@SiO_2_.


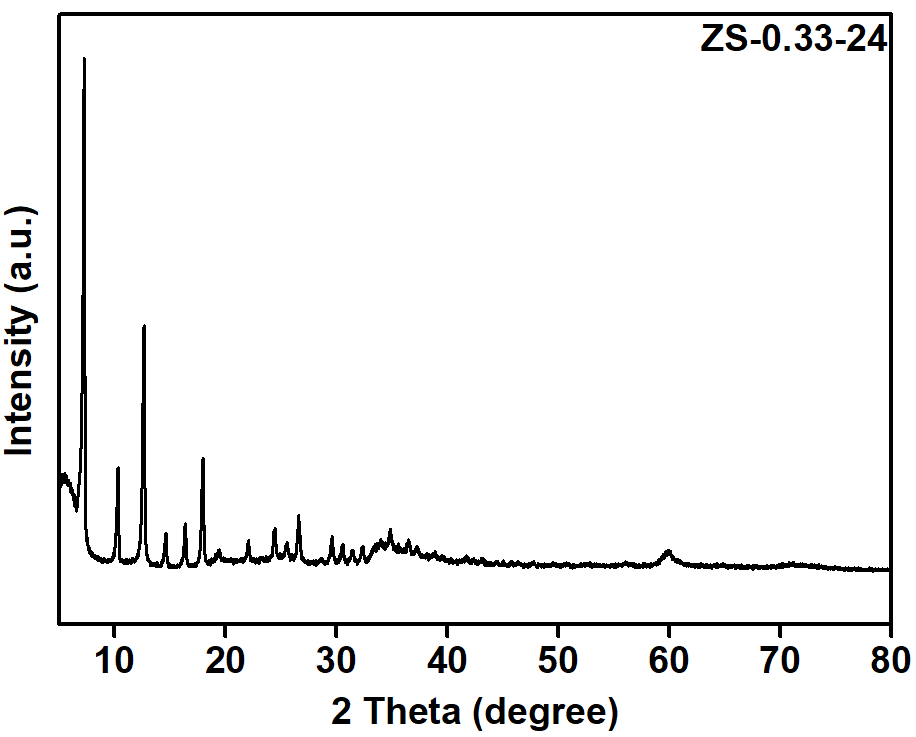


**Figure S6.** XRD pattern of ZS-0.33-24.


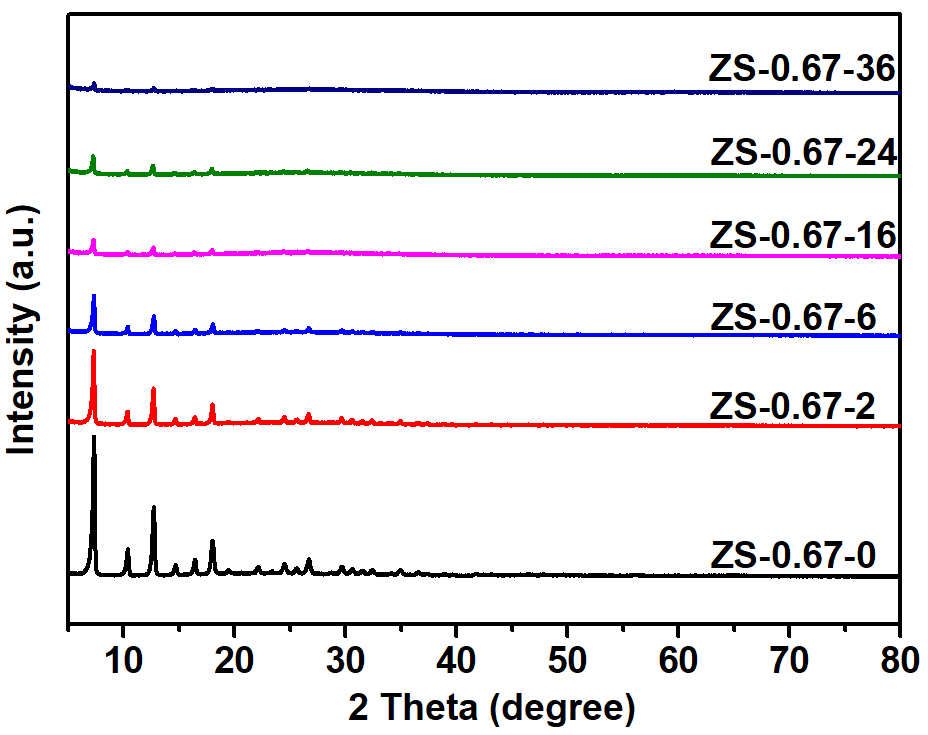


**Figure S7.** XRD patterns of ZS-0.67 with different hydrothermal time.


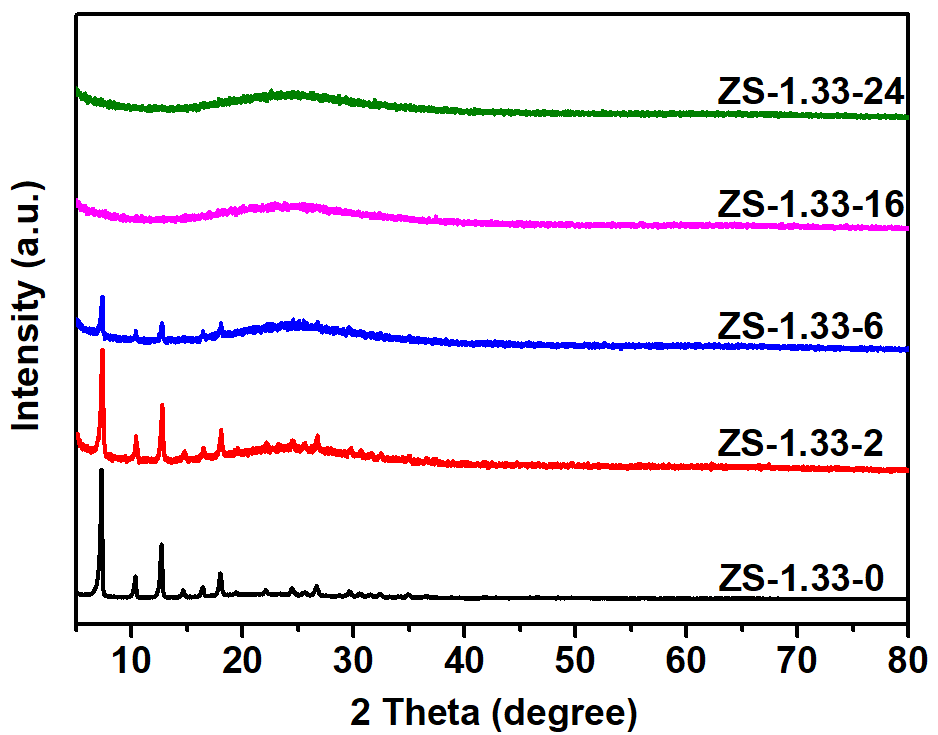


**Figure S8.** XRD patterns of ZS-1.33 with different hydrothermal time.


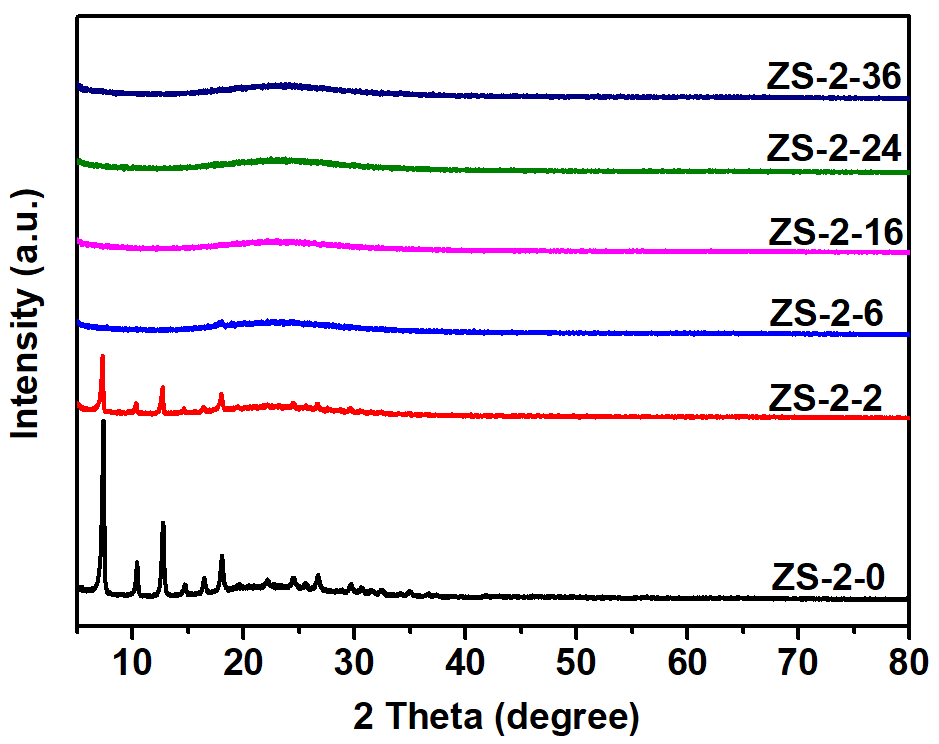


**Figure S9.** XRD patterns of ZS-2 with different hydrothermal time.


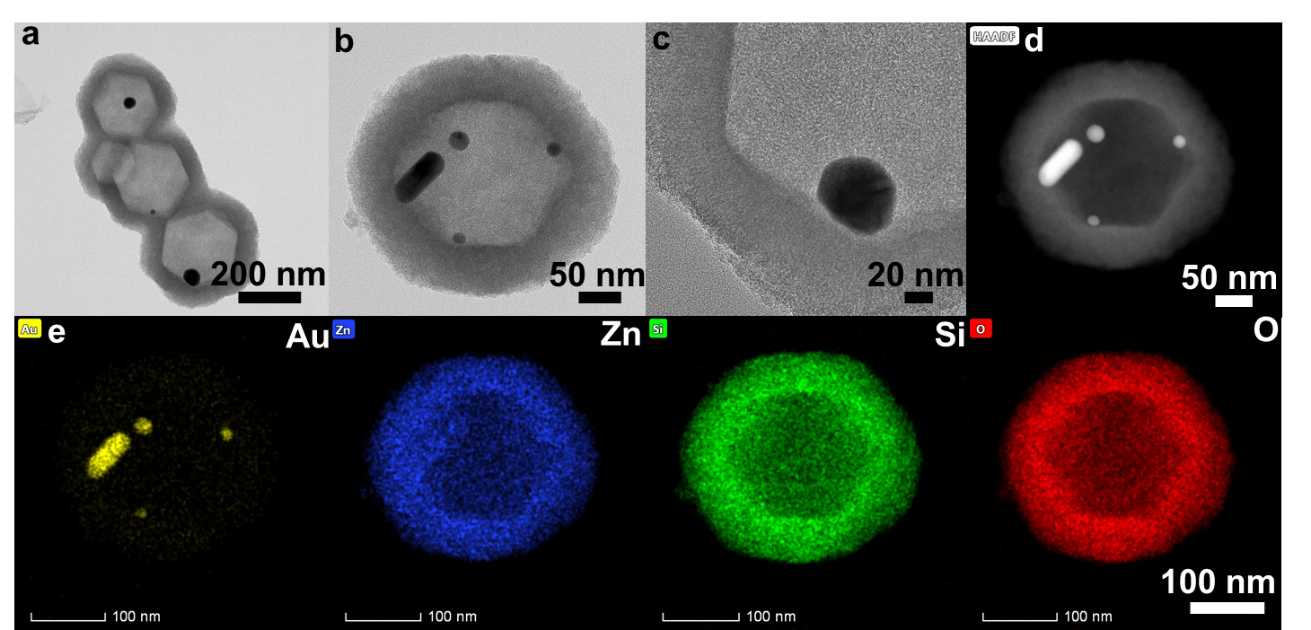


**Figure S10**. (a) (b) (c) TEM images, (d) HAADF image and (e) element mapping images of Au@HMZS.


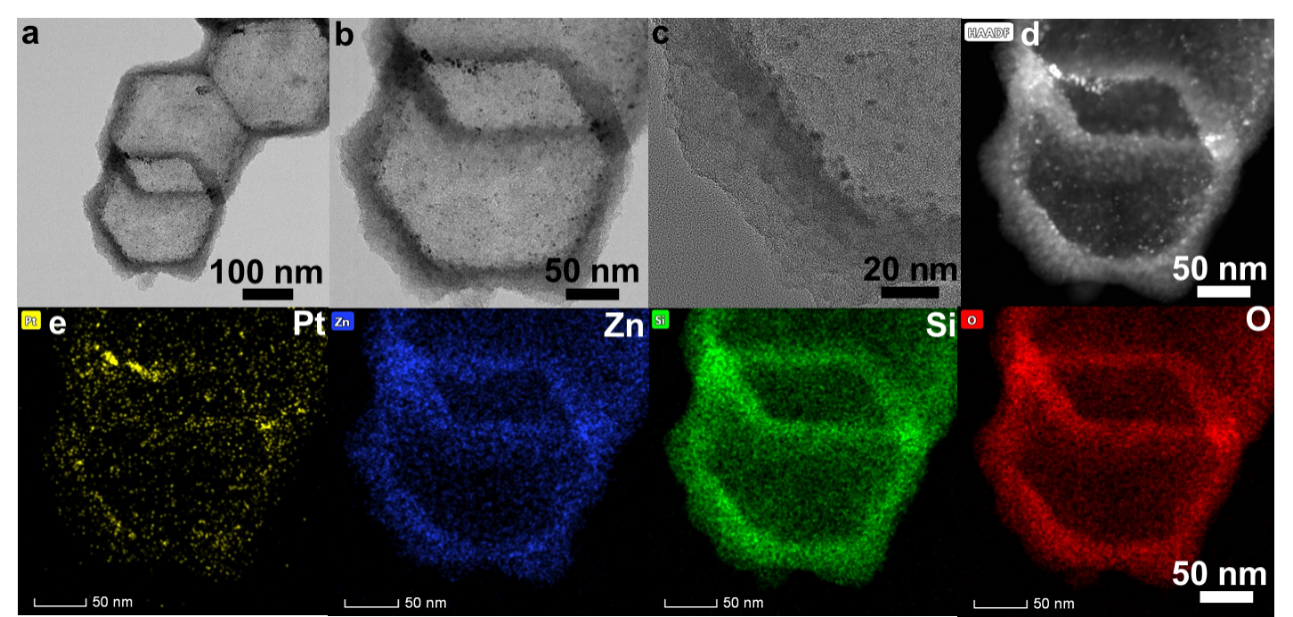


**Figure S11**. (a) (b) (c) TEM images, (d) HAADF image and (e) element mapping images of Pt@HMZS.


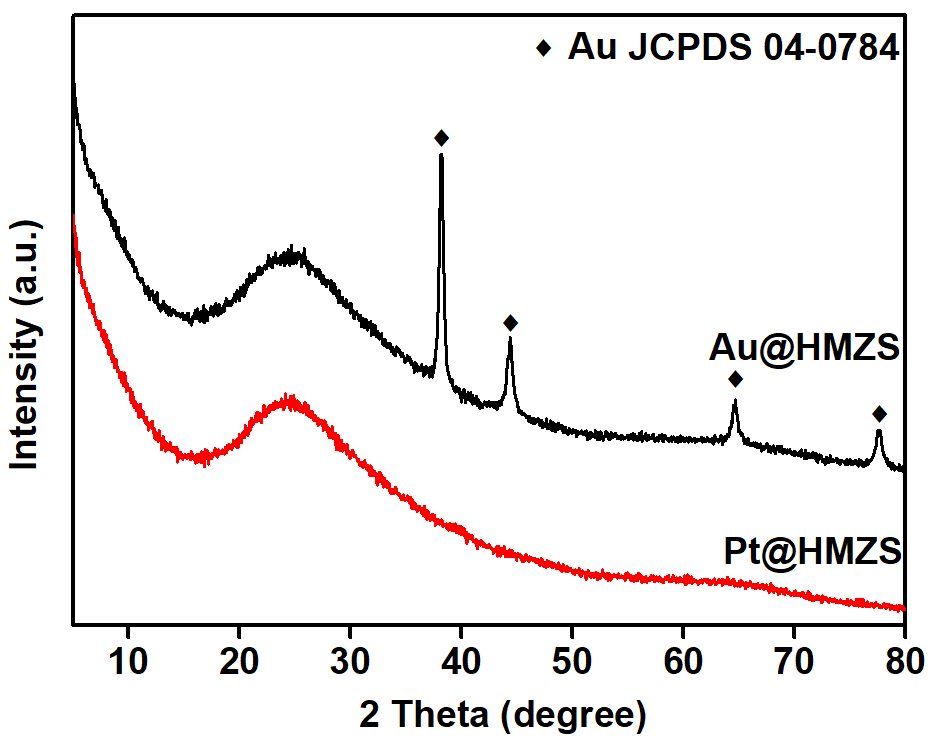


**Figure S12.** XRD patterns of Au@HMZS and Pt@HMZS.


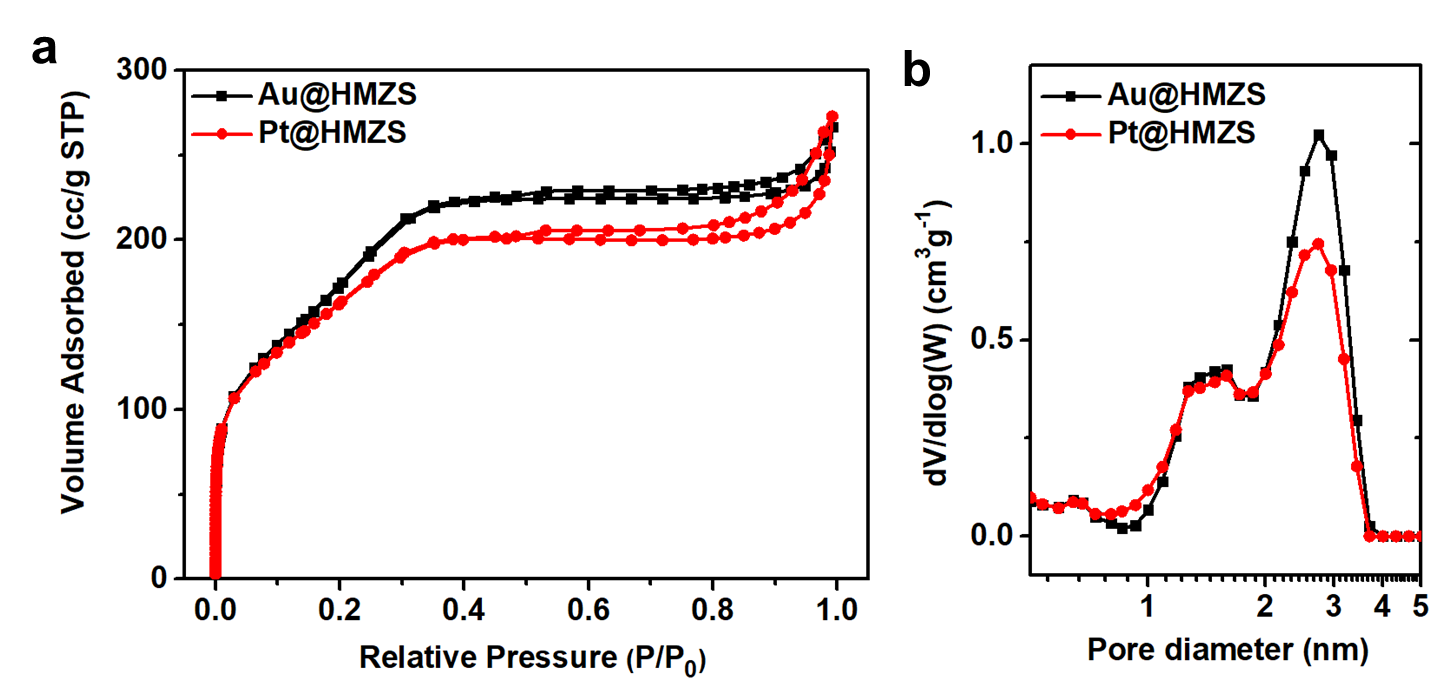


**Figure S13.** N_2_ sorption isotherms (a) and pore size distribution curves (b) of Au@HMZS and Pt@HMZS.


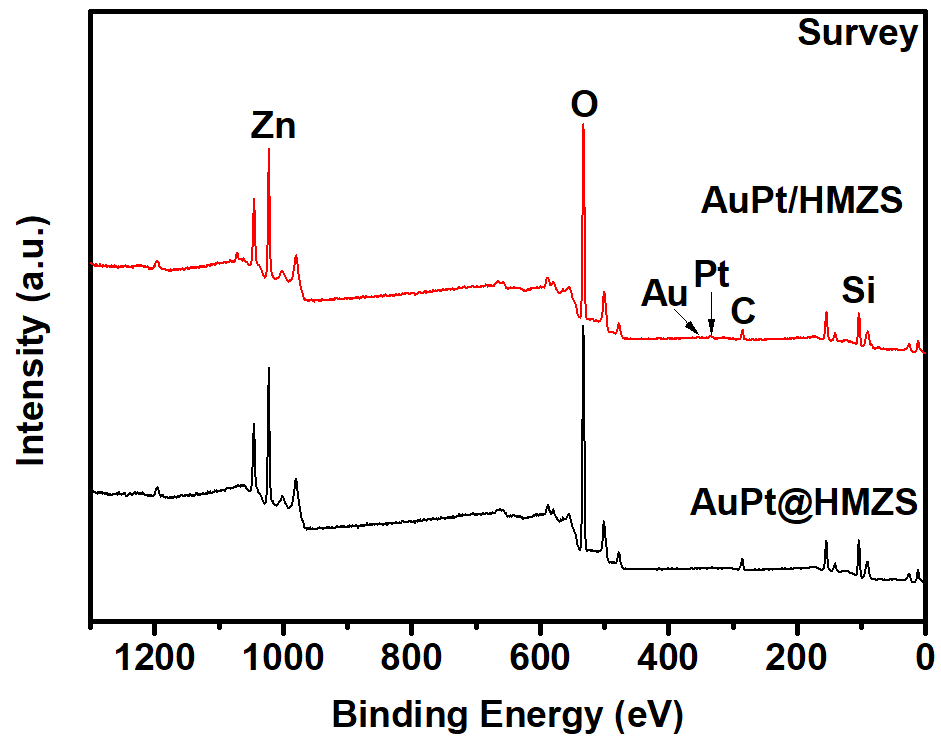


**Figure S14.** XPS survey spectra of AuPt@HMZS and AuPt/HMZS.


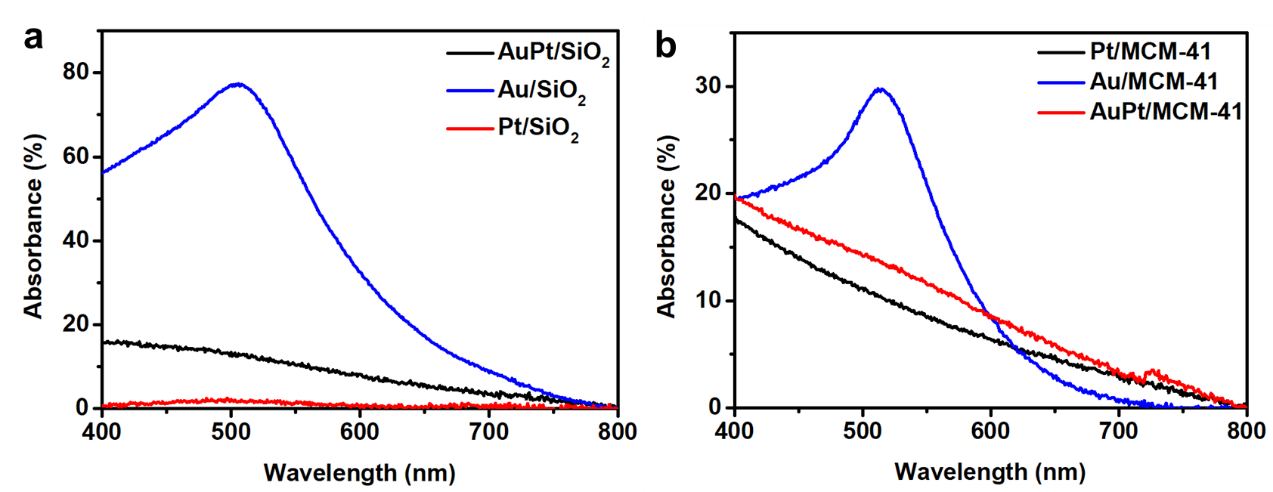


**Figure S15.** UV-Vis spectra of Au/SiO_2_, Pt/SiO_2_ and AuPt/SiO_2_ (a) and Au/MCM-41, Pt/MCM-41 and AuPt/MCM-41 (b).


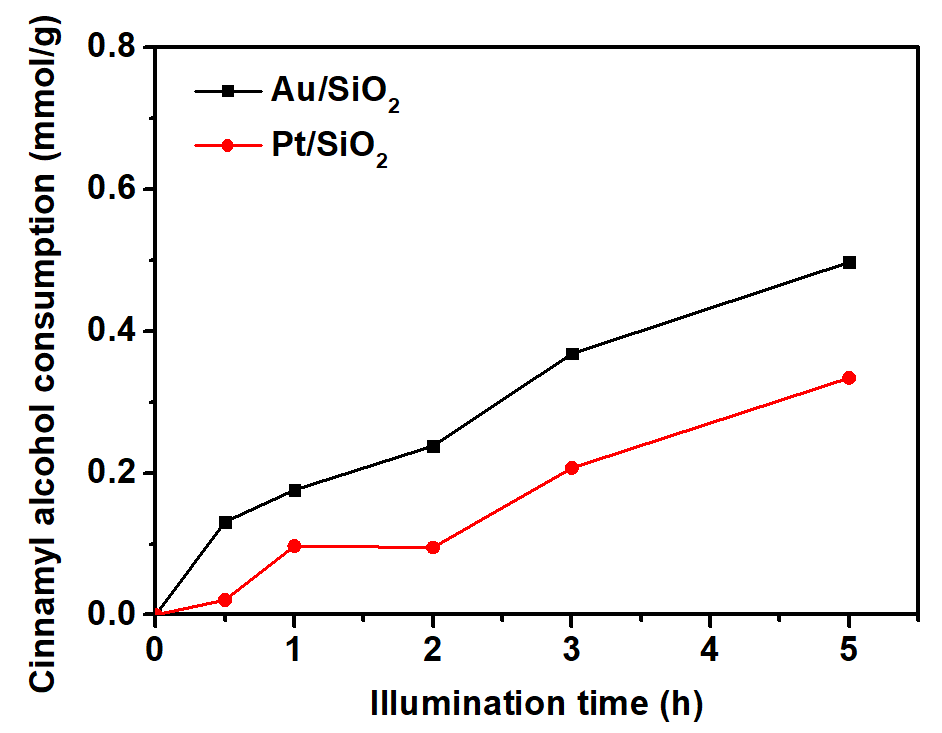


**Figure S16.** Photocatalytic oxidation performance from cinnamyl alcohol solution by using Au/SiO_2_ and Pt/SiO_2_.


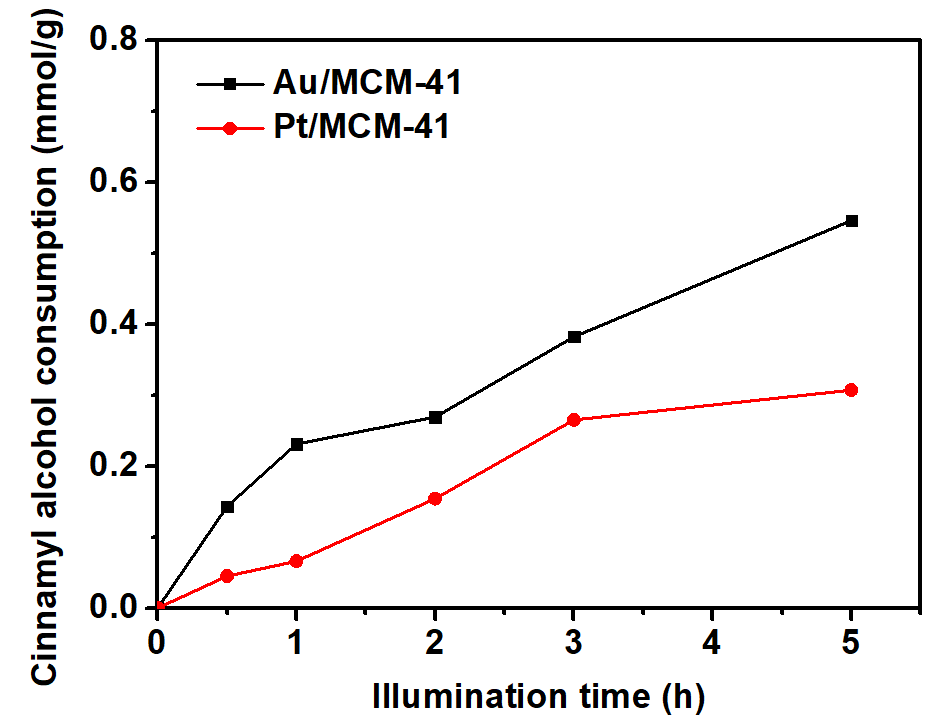


**Figure S17.** Photocatalytic oxidation performance from cinnamyl alcohol solution by using Au/MCM-41 and Pt/MCM-41.

**
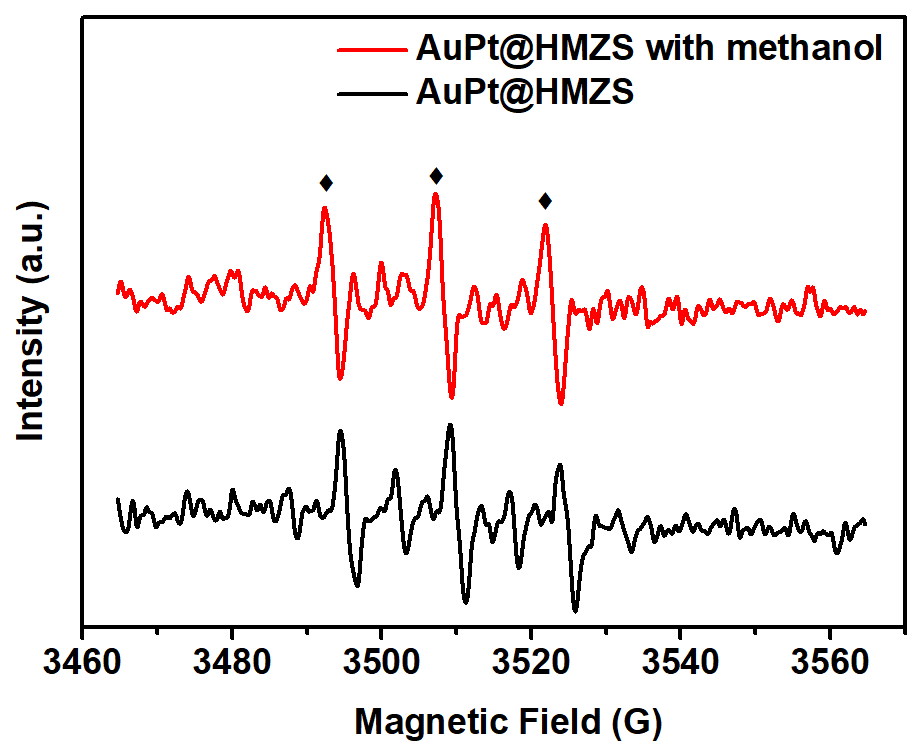
**

**Figure S18.** Electron paramagnetic resonance (EPR) spectra over the AuPt@HMZS catalyst under solar light irradiation.

| **Table S1.** Physical properties of zinc silica composites | | | | | | | | |
| --- | --- | --- | --- | --- | --- | --- | --- | --- |
| Catalysts | S_BET_^a^ (m²/g) | S_micro_^b^ (m²/g) | S_micro_/  S_meso_^b^ | Pore size distribution ^c^ (nm) | V_t_^d^ (cm^3^/g) | V_micro_^e^ (m^3^/g) | V_micro_/V_meso_^f^ | Metal crystalline size^g^ (nm) |
| HMZS-0.67 | 504 | 373 | 3 | 1.4，2.7 | 0.65 | 0.18 | 0.4 | - |
| HMZS-1.33 | 842 | 782 | 13 | 1.4，2.8 | 0.54 | 0.37 | 2.2 | - |
| HMZS-2 | 838 | 796 | 19 | 1.4，2.9 | 0.52 | 0.39 | 3.0 | - |
| Au@HMZS | 666 | 659 | 94 | 1.4，2.7 | 0.41 | 0.34 | 4.8 | 11 |
| Pt@HMZS | 602 | 595 | 85 | 1.4，2.7 | 0.42 | 0.30 | 2.5 | - |

^a^ S_BET_ is the Brunauer-Emmett-Teller (BET) specific surface area.

^b^ S_micro_ is the t-plot-specific micropore surface area calculated from the N_2_ adsorption-desorption isotherm.

^b^ S_meso_ is the specific mesopore surface area estimated by subtracting S_micro_ from S_BET_.

^c^ Pore size distribution is obtained by using DFT method.

^d^ V_t_ is the total specific pore volume determined by using the adsorption branch of the N_2_ isotherm

at P/P_0_=0.99.

^e^ V_micro_ is the t-plot-specific micropore volume calculated from the N_2_ adsorption-desorption isotherm.

^f^ V_meso_ is the specific micropore volume calculated by subtracting V_micro_ from V_t_

^g^ Calculated using the Scherrer equation in the XRD profiles

**Table S2.** The chemical compositions of bimetal zinc silica composites. obtained by XPS characterization.

| Catalysts | C (at.%) | O(at.%) | Si(at.%) | Zn (at.%) | Pt (at.%) | | Au (at.%) |
| --- | --- | --- | --- | --- | --- | --- | --- |
| AuPt/HMZS | 9.33 | 55.62 | 30.92 | 3.84 | 0.13 | 0.16 | |
| AuPt@HMZS | 9.72 | 55.84 | 30.69 | 3.73 | 0.01 | 0.01 | |

| Catalysts | Substrate | Atmosphere | Cocatalysts content (%) | Conversion  (mmol/g/h) | Light source | Light irritation | Solution | Reference |
| --- | --- | --- | --- | --- | --- | --- | --- | --- |
| AuPt@HMZS | 0.1mmol | 2bar O_2_ | 1.5 | 0.62 | 300W Xe lamp | >420nm | water | This work |
| Au/zeolite Y | 3.5mmol | O_2_ atmosphere | 2.3 | 0.31 | 500W Halogen lamp | 400–800 nm | toluene | ^1^ |
| H_3_PW_12_O_40_ (30%)/MCM-41 | 1mmol | O_2_ atmosphere | 30 | 39 | 400W mercury lamp | >300nm | toluene | ^2^ |
| Pt/Fe-STO-600 | 0.3mmol | Ar atmosphere | 3.3 | 0.22 | 300W Xe lamp | >400nm | ionic liquids: [omim]BF4 | ^3^ |
| Cu/Nb_2_O_5_ | 1mmol | 0.1MPa O_2_ | 1.9 | 0.11 | 500 W ultra-high-pressure Hg lamp | >300nm | water | ^4^ |
| BN/In_2_S_3_ | 0.5mmol | 0.1MPa O_2_ | 7 | 0.53 | 300W Xe lamp | >420nm | benzene | ^5^ |
| Pt/WO_3_ | 1.5mmol | O_2_ atmosphere | 1.5 | 0.41 | 230 W tungsten-halogen lamp | >420nm | benzotrifluoride | ^6^ |
| Pd-BiOCl | 0.05mmol | O_2_ atmosphere | 1.5 | 2.93 | 300W Xe lamp | 360-780nm | water | ^7^ |

**Table S3.** Comparison of catalytic activities of photocatalysts loaded with co-catalysts.

**References:**

1. Zhang, X.; Ke, X.; Zhu, H., Zeolite-Supported Gold Nanoparticles for Selective Photooxidation of Aromatic Alcohols under Visible-Light Irradiation. *Chem. Eur. J.* **2012,** *18* (26), 8048-8056.

2. Shen, H.-Y.; Mao, H.-L.; Ying, L.-Y.; Xia, Q.-H., Photocatalytic selective aerobic oxidation of alcohols to aldehydes and ketones by HPW/MCM-41 in ionic liquids. *J. Mol. Catal. A: Chem.* **2007,** *276* (1), 73-79.

3. Hu, Y.; Zhao, G.; Pan, Q.; Wang, H.; Shen, Z.; Peng, B.; Busser, G. W.; Wang, X.; Muhler, M., Highly Selective Anaerobic Oxidation of Alcohols Over Fe-doped SrTiO3 Under Visible Light. *ChemCatChem* **2019,** *11* (20), 5139-5144.

4. Furukawa, S.; Tamura, A.; Shishido, T.; Teramura, K.; Tanaka, T., Solvent-free aerobic alcohol oxidation using Cu/Nb_2_O_5_: Green and highly selective photocatalytic system. *Appl. Catal. B* **2011,** *110*, 216-220.

5. Meng, S.; Ye, X.; Ning, X.; Xie, M.; Fu, X.; Chen, S., Selective oxidation of aromatic alcohols to aromatic aldehydes by BN/metal sulfide with enhanced photocatalytic activity. *Appl. Catal. B* **2016,** *182*, 356-368.

6. Qamar, M.; Fawakhiry, M. O.; Azad, A. M.; Ahmed, M. I.; Khan, A.; Saleh, T. A., Selective photocatalytic oxidation of aromatic alcohols into aldehydes by tungsten blue oxide (TBO) anchored with Pt nanoparticles. *RSC Adv.* **2016,** *6* (75), 71108-71116.

7. Li, B.; Shao, L.; Wang, R.; Dong, X.; Zhao, F.; Gao, P.; Li, Z., Interfacial synergism of Pd-decorated BiOCl ultrathin nanosheets for the selective oxidation of aromatic alcohols. *J. Mater. Chem. A* **2018,** *6* (15), 6344-6355.
